# Comparison of Selected Parameters of Redox Homeostasis in Patients with Ataxia-Telangiectasia and Nijmegen Breakage Syndrome

**DOI:** 10.1155/2017/6745840

**Published:** 2017-12-31

**Authors:** Barbara Pietrucha, Edyta Heropolitanska-Pliszka, Mateusz Maciejczyk, Halina Car, Jolanta Sawicka-Powierza, Radosław Motkowski, Joanna Karpinska, Marta Hryniewicka, Anna Zalewska, Malgorzata Pac, Beata Wolska-Kusnierz, Ewa Bernatowska, Bozena Mikoluc

**Affiliations:** ^1^Clinical Immunology, The Children's Memorial Health Institute, Av. Dzieci Polskich 20, 04-730 Warsaw, Poland; ^2^Department of Experimental Pharmacology, Medical University of Bialystok, Szpitalna 37 Str., 15-295 Bialystok, Poland; ^3^Department of Family Medicine, Medical University of Bialystok, Bialystok, Poland; ^4^Department of Pediatrics Rheumatology, Immunology, and Metabolic Bone Diseases, Medical University of Bialystok, Waszyngtona 17 Str., 15-274 Bialystok, Poland; ^5^Institute of Chemistry, University of Bialystok, Bialystok, Poland; ^6^Department of Conservative Dentistry, Medical University of Bialystok, Bialystok, Poland

## Abstract

This study compared the antioxidant status and major lipophilic antioxidants in patients with ataxia-telangiectasia (AT) and Nijmegen breakage syndrome (NBS). Total antioxidant status (TAS), total oxidant status (TOS), oxidative stress index (OSI), and concentrations of coenzyme Q10 (CoQ10) and vitamins A and E were estimated in the plasma of 22 patients with AT, 12 children with NBS, and the healthy controls. In AT patients, TAS (median 261.7 *μ*mol/L) was statistically lower but TOS (496.8 *μ*mol/L) was significantly elevated in comparison with the healthy group (312.7 *μ*mol/L and 311.2 *μ*mol/L, resp.). Tocopherol (0.8 *μ*g/mL) and CoQ10 (0.1 *μ*g/mL) were reduced in AT patients versus control (1.4 *μ*g/mL and 0.3 *μ*g/mL, resp.). NBS patients also displayed statistically lower TAS levels (290.3 *μ*mol/L), while TOS (404.8 *μ*mol/L) was comparable to the controls. We found that in NBS patients retinol concentration (0.1 *μ*g/mL) was highly elevated and CoQ10 (0.1 *μ*g/mL) was significantly lower in comparison with those in the healthy group. Our study confirms disturbances in redox homeostasis in AT and NBS patients and indicates a need for diagnosing oxidative stress in those cases as a potential disease biomarker. Decreased CoQ10 concentration found in NBS and AT indicates a need for possible supplementation.

## 1. Introduction

Ataxia-telangiectasia (AT; OMIM #208900) and Nijmegen breakage syndrome (NBS; OMIM #251260) belong to a group of genetically determined primary immunodeficiency disorders (PID) whose course involves defects in DNA repair processes [1]. AT is caused by null mutations in the *ATM* (*ataxia-telangiectasia mutated*) *gene* located on chromosome 11q.26 which encodes a protein of the same name. The main role of ATM protein is the coordination of cellular response to DNA double strand breaks, oxidative stress, signal transduction, and cell-cycle control. The clinical picture of the disease is characterised by cerebellar degeneration, telangiectasia, immunodeficiency, cancer susceptibility, and radiation sensitivity. Experimental studies have provided direct evidence for the presence of mitochondrial dysfunctions in AT cells. They have demonstrated that the structural organisation of mitochondria in AT cells is abnormal compared to wild-type cells [2, 3].

NBS, similarly to AT, belongs to a group of the XCIND syndrome. The XCIND syndrome is named after Eaton et al. who found distinct hypersensitivity to ionizing (X-ray) irradiation, cancer susceptibility, immunodeficiency, neurological abnormality, and double-strand DNA breakage in these cases [4]. Mutations causative of NBS occur in the NBS1 gene, located on human chromosome 8q21. NBS1 encodes for nibrin, the key regulatory protein of the R/M/N (RAD50/MRE11/NBS1) protein complex which senses and mediates cellular response to DNA damage caused by ionizing radiation [5, 6]. The chromosome instability in AT and NBS patients results from a defective response to DNA double-strand breaks. In addition, AT and NBS patients display a few common characteristics such as growth retardation, premature aging, and neurodegeneration. It is believed that these symptoms can be caused not only by chromosomal instability and aberrant DNA damage response but also by oxidative stress and mitochondrial abnormalities [1, 2].

Recent studies indicate new, alternative sources of ROS and oxidative stress in AT and NBS cells, including NADPH oxidase 4, oxidised low-density lipoprotein (ox-LDL), or poly (ADP-ribose) polymerases (PARP-1, PARP-2, and PARP-3) [1, 7–10]. Mitochondrial dysfunction such as aberrant structural organisation of mitochondria, excess mitochondrial ROS (mROS) production and mitochondrial injury have also been reported in AT and NBS cells [1, 3, 11]. Valentin-Vega et al. [11] showed increased mROS production in AT cells, which resulted from a decline in the activity of complex I of the electron transport chain in mitochondria. Noteworthy is a study conducted by Weyemi et al. [9] who stressed that NADPH oxidase 4 (the main source of free radicals in the cell) was highly upregulated in AT cells and correlated with higher oxidative damage and apoptosis. To date, however, little is still known about the oxidant/antioxidant abnormalities in AT and NBS patients. Therefore, the purpose of our study was to measure selected parameters of redox homeostasis in patients with AT and NBS. Both disorders are characterised by symptoms typical of the XCIND syndrome, and therefore we intended to evaluate total antioxidant status (TAS), total oxidant status (TOS), and oxidative stress index (OSI) as well as concentrations of the most common lipophilic antioxidants, CoQ10, and vitamins A and E. It appears that the thorough understanding of disturbances in the body's antioxidant defense mechanisms could lead to new therapeutic strategies in AT and NBS, similarly as in other oxidative stress-related genetic disorders.

## 2. Materials and Methods

### 2.1. Patients

The study included 12 Caucasian children with NBS (5 females, 7 males) whose average age was 12 years and 1 month with a confirmed mutation in the *NBS1 gene* and 22 patients with AT (9 females, 13 males) whose average age was 13 years and 7 months with a confirmed mutation in *ATM gene*. All study participants were under the medical care of the Department of Immunology at the Children's Memorial Health Institute in Warsaw, Poland. The patients were included in the study on the basis of their medical history and physical examination, and they were found to be in good health at the time of enrollment (normal biochemical and morphological blood parameters, negative markers of inflammation). Diagnosis was based on clinical symptoms and genetic and biochemical tests according to ESID (European Society for Immunodeficiencies) criteria. None of the patients enrolled in the study were diagnosed with cancer, diabetes, hypertension, and HIV infection. The control group consisted of, respectively, 12 and 22 healthy individuals matched for age and sex. Plasma samples of the study and the control group were collected between February 2016 and January 2017.

The study was approved by the Bioethical Committee at the Medical University, Bialystok, Poland. Parents of all respondents gave informed written consent for their children's participation in the study.

### 2.2. Determination of Plasma Total Antioxidant Status (TAS), Total Oxidant Status (TOS), and Oxidative Stress Index (OSI)

Total antioxidant status (TAS) and total oxidant status (TOS) were determined using commercial colorimetric kits (ImAnOx (TAS/TAC) Kit, Immundiagnostik, Bensheim, Germany and PerOx (TOS/TOC) Kit, Immundiagnostik, Bensheim, Germany, resp.) in accordance with the manufacturer's instructions. The determination of TAS was based on a reaction between antioxidants contained in the sample with exogenous hydrogen peroxide, while the determination of TOS was performed by the reaction of peroxidase with total lipid peroxides in the sample. The resulting coloured products were measured colorimetrically at a wavelength of 450 nm using Mindray MR-96 Microplate Reader, China. All assays were performed in duplicate samples. To assess the redox balance disorders, we have also used the oxidative stress index (OSI), which may be considered as a gold indicator of oxidative stress in biological systems. OSI was calculated according to the formula OSI = TOS/TAS × 100 [12].

### 2.3. Determination of Plasma Vitamin A (All-Trans-Retinol), Vitamin E (L-Tocopherol), and Coenzyme Q10

Analyses were performed using high performance liquid chromatograph coupled with MS detector equipped with triple quadrupole (Shimadzu LCMS/MS-8040). Ionization was conducted using APCI (atmospheric pressure chemical ionization) mode. Data acquisition and processing were performed using Shimadzu LabSolutions LCMS Software.

The compounds were separated with a Kinetex XB-C18 100A analytical column (50 mm × 3.0 mm, 1.7 *μ*m). The mobile phase consisted of an isocratic solvent A (methanol) 0.01-2 min and then isocratic solvent B (methanol-n-hexan, 72 : 28, *v*/*v*) 2.5–6 min. The flow rate was 0.4 mL/min, and the temperature of the analytical column was 400°C. The injection volumes of standard and sample solutions were 10 *μ*L. Acquisition settings and method were optimized by the infusion of a 10 *μ*g/mL solution of each fixed compound. The mass spectrometer was operated in the positive ion atmospheric pressure chemical ionization mode. The APCI temperature was set at 350°C and the ion current 4.5 *μ*A. The flow of the drying gas (N2) and the flow of the nebulizing gas were 10 L/min and 3 L/min, respectively. The desolvation line (DL) and heat block temperature was 230°C.

**Figure 1:**
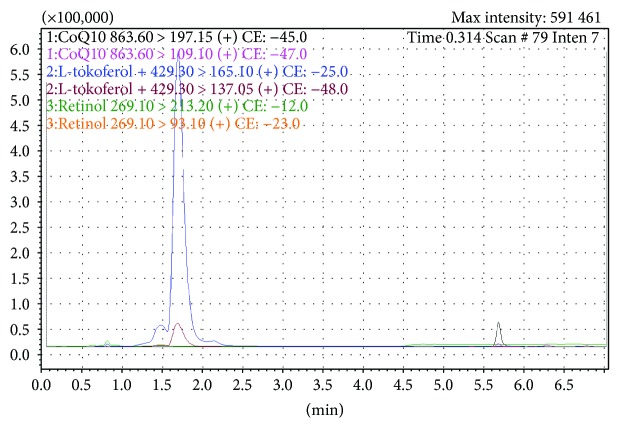
Chromatogram of a real sample (plasma).

All analytes were detected in the MS/MS multiple reaction monitoring (MRM) with unit resolution at both Q1 and Q3. The MS conditions for generation of the positive ions are presented in Table 1. The described above chromatographic conditions were used for quantification of target analytes in plasma samples. The chromatogram of real sample is visualized in Figure 1. Plasma samples were prepared according to the procedure published previously [13].

### 2.4. Statistical Analysis

The examined variable distribution was assessed by means of the Kolmogoroff-Smirnow test. Due to the fact that the tested variables were inconsistent with normal distribution, the Mann–Whitney *U* test was used. Results are expressed as median, minimum, and maximum. The Spearman's method was applied in assessing correlations between variables. In calculations, the relevance level of *p* < 0.05 was accepted as statistically significant, authorising the rejection of individual zero hypotheses. The data were processed using the Polish version of Statistica 12.0 statistical software for PC with Windows.

## 3. Results

**Figure 2:**
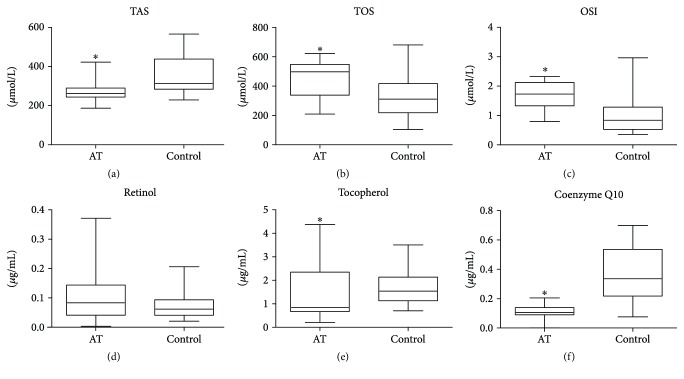
Lipophilic antioxidants and total antioxidant/oxidant status in patients with AT and control group. TAS: total antioxidant status; TOS: total oxidant status; OSI: oxidative stress index; ^∗^*p* < 0.05.

Our study demonstrated that in patients with AT plasma TAS levels were statistically lower (*p* = 0.002) and plasma TOS was statistically significantly elevated (*p* = 0.001) in comparison with the control group. Similarly to TOS, OSI was elevated in AT patients (*p* = 0.001) (Figure 2).

Tocopherol plasma concentrations were significantly reduced in AT patients as compared to the control group (*p* = 0.021). The concentrations of endogenous free radical scavenger coenzyme Q10 were statistically decreased in the plasma of AT patients versus the healthy controls (*p* = 0.001). There were no significant differences in the concentrations of plasma retinol between both groups (*p* = 0.076) (Figure 2).

**Figure 3:**
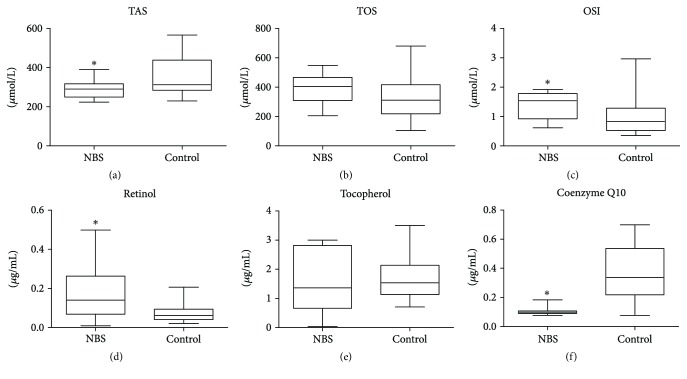
Lipophilic antioxidants and total antioxidant/oxidant status in patients with NBS and control group. TAS: total antioxidant status; TOS: total oxidant status; OSI: oxidative stress index; ^∗^*p* < 0.05.

Similarly to the AT group, NBS patients displayed statistically lower plasma TAS levels in comparison with the control group (*p* = 0.044). In contrast to AT, plasma TOS in NBS was not significantly different in comparison with the healthy controls (*p* = 0.102). The OSI value, similarly to AT, was higher in comparison with the controls (*p* = 0.004) (Figure 3).

We demonstrated that in patients with NBS the concentration of vitamin A precursor, beta carotene, was significantly elevated in comparison with that in the control group (*p* = 0.011). The difference in CoQ10 concentration in NBS patients and healthy controls points to a significantly lower level in NBS patients (*p* = 0.001). The concentrations of plasma vitamin E were similar in the control group and NBS patients (*p* = 0.582) (Figure 3).

It is worth noting that we did not observe statistically significant differences between the evaluated parameters in patients with AT and those with NBS (Table 2). In addition, we did not find significant correlations in the assessed markers of oxidative stress between the study groups and controls.

## 4. Discussion

**Figure 4:**
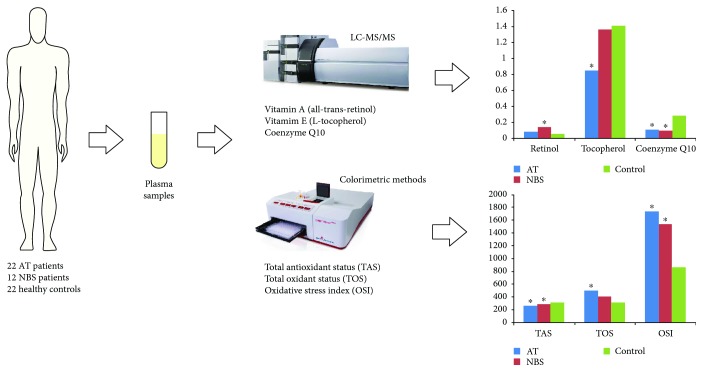
Graphical presentation of the study. ^∗^*p* < 0.05 versus control.

This study compared the redox balance as well as major lipophilic antioxidants in patients with AT and NBS diseases. We have shown that AT and NBS are associated with impaired redox homeostasis including disturbances in lipophilic free radical scavengers such as coenzyme Q10 and alpha-tocopherol (Figure 4).

We aimed to investigate the oxidant/antioxidant status via the measurement of plasma TAS and TOS, which could provide a systemic reflection of redox homeostasis in biological systems. Concentration of TAS was significantly reduced in AT and NBS patients, which suggests the exhaustion or inefficiency of protective antioxidant systems resulting from the overproduction or enhanced activity of reactive oxygen species (ROS). It is believed that oxidative stress plays a key role in the development of many systemic complications including growth retardation, endocrine abnormalities, neurodegeneration, and premature aging as well as immunodeficiency [14–16], which are the major phenotypic hallmarks in AT and NBS diseases [1]. Our study did not directly demonstrate oxidative stress in patients with AT and NBS, since it would have been necessary to measure oxidative modification products. Nevertheless, we showed the existence of cellular redox abnormalities in the AT (↓ TAS, ↑ OSI, and ↑ TOS) and NBS groups (↓ TAS and ↑ OSI), which can constitute the basis for the development of oxidative stress leading to DNA mutations and protein oxidation as well as lipid peroxidation. We would like to emphasise that we did not find significant differences between the researched parameters of redox balance when we compared AT with NBS. A lack of correlation may indicate a similarity of redox imbalance in AT and NBS disorders, although these changes tend to be more severe in patients with AT. However, we recorded significantly elevated TOS levels only in patients with AT and therefore, TOS may be subtle distinguishing between AT and NBS diseases.

There is a consensus in the literature regarding enhanced oxidative stress in neurodegenerative disorders such as Alzheimer's or Parkinson's disease (PD) [17, 18]. Clinical studies have confirmed increased oxidant status and comparable, decreased antioxidant status in patients with PD [19–21]. It is probable that similar changes occur in the course of AT and NBS. However, the available literature contains limited data regarding oxidative stress/redox disturbances in those cases [1]. Additionally, it should be noted that the measurement of plasma TAS and TOS provides information about antioxidant properties conditioned primarily by low molecular weight (LWMA) hydrophilic antioxidants (e.g., uric acid (UA) and ascorbic acid (AA) as well as thiol groups), but not lipophilic antioxidants [22]. Taking this into consideration, we also decided to measure levels of major lipophilic free radical scavengers (CoQ10, vitamins A and E).

Our study confirm decreased CoQ10 concentration both in AT and NBS. Coenzyme Q10 (also called ubiquinone) is a lipid soluble benzoquinone which is a key component of the mitochondrial respiratory chain for adenosine triphosphate (ATP) synthesis [23]. It has been demonstrated that most of the CoQ10 in the human body is produced endogenously; 25% of the stores are obtained from dietary intake [24]. Coenzyme Q10 deficiency results not only in abnormal respiratory chain function with inadequate cellular energy production, increased generation of free radicals, and degradation of mitochondria but also impacts on the immune system [25]. Literature reports demonstrate that CoQ10 supplementation has improved CD4 T cell counts in patients with AIDS and outcomes in herpes and HPV infections. Furthermore, leukocyte activity is conditioned, inter alia, by proper CoQ10 activity [25]. CoQ10 deficiency may contribute to the abnormal function of mitochondria in the course of diseases such as AIDS, Alzheimer's disease, Parkinson's disease, and cancer [26, 27]. Similar disturbances have been observed in AT and NBS cells, which may suggest a potential involvement of CoQ10 in the pathogenesis of these disorders [1]. Considering the protective action of CoQ10 in neurodegenerative processes and its role in the immune system, decreased CoQ10 concentration in AT and NBS patients indicates a need for monitoring its concentration and potential supplementation. At this stage, we are unable to answer the question to what degree clinical symptoms observed in AT, such as cerebellar degeneration, immunodeficiency, cancer susceptibility, and radiation sensitivity, result from oxidative stress disturbances observed in our study. Are these disturbances primary or secondary? However, they undeniably confirm CoQ10 involvement in the pathomechanism of the observed changes. It should be also emphasised that rare autosomal recessive disorder, CoQ10 deficiency (mutation in CABC1; COQ2; COQ9; PDSS1; PDSS2 genes), is frequently associated with seizures, cognitive decline, pyramidal track signs, myopathy, and prominent cerebellar ataxia [28, 29]. Some of these symptoms occur in both AT and NBS. Additionally, it is very likely that, in AT and NBS patients, a decrease in CoQ10 levels may be associated with impaired DNA repair mechanisms, similarly as in other bioenergetics disorders such as xeroderma pigmentosum (XP), Cockayne Syndrome (CS), Fanconi anaemia (FA), and Hutchinson-Gilford syndrome (HGS) [1].

Deficiency in lipid soluble antioxidants has been demonstrated in various conditions such as eating disorders, nicotine addiction, chronic diseases, and aging. Antioxidant vitamins and trace elements contribute to maintaining an effective immune response [30–33]. Treatments are also available for a few autosomal recessive ataxias: vitamin E therapy for ataxia with vitamin E deficiency (AVED), chenodeoxycholic acid for cerebrotendinous xanthomatosis, CoQ10 for CoQ10 deficiency, and dietary restriction of phytanic acid for Refsum disease. Idebenone may ameliorate the cardiac and neurological manifestations of Friedreich ataxia (FRDA). No other specific treatments exist for hereditary ataxias [34]. Unfortunately, no sufficiently effective causal therapy is available for AT and NBS patients at present. Our results indicate a need for introducing CoQ10 and tocopherol into AT and NBS treatment, taking into consideration their plasma levels.

Alpha-tocopherol primarily inhibits the production of new free radicals and thus might help in preventing or delaying chronic diseases associated with reactive oxygen species molecules [35]. Decreased vitamin E concentration in AT may result in a decline in the body's antioxidant activity and anti-inflammatory as well as cell-mediated and humoral immune functions. In AT cases, we found decreased concentrations of both CoQ10 and vitamin E, which enhance disturbances in the redox balance and may increase ROS production, accumulation of damaged mitochondrial DNA (mDNA), and progressive respiratory chain dysfunction. The few experimental studies found in the available literature report that disturbances in redox processes also occur in NBS [1]. Our study of NBS cases demonstrated decreased TAS and enhanced OSI on the one hand and a reduced concentration of CoQ10 and increased concentration of vitamin A, on the other hand. Despite the fact that the interpretation of obtained results is difficult at this stage of research advancement due to a lack of comparable studies, we would like to emphasise that we did not find any differences in the tested parameters between AT and NBS.

To date, only few studies have evaluated the antioxidant defenses in AT and NBS patients [1, 36]. In one of the first reports on the subject, Reichenbach et al. showed a decrease in the levels of total antioxidant capacity, vitamin A, and vitamin E in AT patients. In contrast to our study, CoQ10 levels in AT were not significantly reduced [37]. In our study patients with AT, we found not only significantly reduced levels of CoQ10 but also of *α*-tocopherol. However, retinol concentration, in line with studies by Da Silva et al., was similar to the control group [38]. It is probable that the aforementioned dissimilarities result from differences in the size of study groups and research methodologies. At this point, it should be emphasized that our study is the first in which the largest group of AT and NBS patients took part.

In summary, we demonstrated a significant decrease in the level of plasma TAS in AT and NBS patients as well as an increase in the oxidative stress index. It appears that disturbances in the body's antioxidant defense mechanisms may lead to oxidative damage and altered cellular redox homeostasis and thus affect clinical manifestations of AT and NBS phenotypes. Redox imbalance in patients with AT and NBS appears to exist at a comparable level. However, a significant increase in total oxidant status (TOS) was demonstrated only in patients with AT in whom plasma TOS may be a subtle distinguishing feature of AT and NBS disorders. We are considering the theory that antioxidant supplementation with tocopherol and CoQ10 may be one of the factors enhancing treatment efficacy in AT and NBS diseases and improving patients' clinical status. Further studies regarding CoQ10 in AT and NBS are needed in order to assess its concentration not only in the blood but also in selected tissues and to evaluate the dynamics of change in relation to disease duration. The issue of a possible link between CoQ10 deficiency and the occurrence of mutations responsible for CoQ10 deficiency remains open.

When analyzing the results of our research, one must bear in mind its limitations. We evaluated only selected parameters of redox balance in patients with AT and NBS, so determination of other markers may lead to other conclusions and clinical applications. Additionally, blood oxidative stress biomarkers cannot be mechanistically informative for some reasons. Firstly, blood and the respective tissue under study do not share common redox components and/or pathways (e.g., lack of mitochondria in erythrocytes and plasma). Secondly, both plasma and blood cells can autonomously produce significant amounts of ROS [15, 16]. However, it should be also emphasized that extremely strong point of our study is the large number of patients in the study groups compared to previously published works. Our research may also provide a new practical aspects for clinicians.

## 5. Conclusions

1. Our study confirms disturbances in redox homeostasis in AT and NBS patients and indicates a need for diagnosing oxidative stress in those cases as a potential disease biomarker.
2. The statistically significantly decreased coenzyme Q10 concentration found in NBS and AT indicates a need for possible supplementation aimed at remedying the diagnosed deficiencies in those individuals.

## Figures and Tables

**Table 1:** The MS conditions for generation of positive ions of the analytes.

Compound	Precursor ion (m/z)	Product ions (m/z)	Collision energy [eV]
Retinol	269.10	213.20 93.10	−12 −23

*α*-Tocopherol	429.30	165.10 137.05	−25 −48

Coenzyme Q10	863.60	197.15 109.10	−45 −47

m/z: mass-to-charge ratio.

**Table 2:** Differences between lipophilic antioxidants and total antioxidant/oxidant status in patients with AT and NBS.

Parameters examined	AT group		NBS group		*p*
	Median	Min–max	Median	Min–max	
Retinol (*μ*g/mL)	0.083	0.003–0.371	0.140	0.009–0.498	0.109
*α*-Tocopherol (*μ*g/mL)	0.847	0.213–4.371	1.361	0.032–3.003	0.929
Coenzyme Q10 (*μ*g/mL)	0.108	0.085–0.206	0.098	0.077–0.183	0.290
TAS (*μ*mol/L)	261.745	186.852–422.515	290.288	223.966–389.895	0.327
TOS (*μ*mol/L)	496.839	209.803–621.354	404.795	204.741–548.632	0.094
OSI	1.730	0.790–2.320	1.543	0.615–1.915	0.157

TAS: total antioxidant status; TOS: total oxidant status; OSI: oxidative stress index.

## Abbreviations

APCI: Atmospheric pressure chemical ionization
AT: Ataxia-telangiectasia
ATM: Ataxia-telangiectasia mutated
CoQ10: Coenzyme Q10
ESID: European Society for Immunodeficiencies
LWMA: Low molecular weight hydrophilic antioxidants
MRM: MS/MS multiple reaction monitoring
NBS: Nijmegen breakage syndrome
OSI: Oxidative stress index
PARP: Poly (ADP-ribose) polymerase
PID: Primary immunodeficiency disorder
ROS: Reactive oxygen species
TAS: Total antioxidant status
TOS: Total oxidant status
XCIND syndrome: Hypersensitivity to ionizing (X-ray) irradiation, cancer susceptibility, immunodeficiency, neurological abnormality, and double-strand DNA breakage.
